# Heterotopic Ossification of the Inferior Vena Cava Wall: A Case Report and Literature Review

**DOI:** 10.3389/fsurg.2021.738934

**Published:** 2021-12-02

**Authors:** Jihua Tian, Li Zhang, Min Hu, Xing Zeng, Yongjun Wang, Chunguang Yang, Zhiquan Hu

**Affiliations:** ^1^Department of Urology, Tongji Hospital, Tongji Medical College, Huazhong University of Science and Technology, Wuhan, China; ^2^Department of Cardiovascular Surgery, Tongji Hospital, Tongji Medical College, Huazhong University of Science and Technology, Wuhan, China; ^3^Department of Cardiovascular Surgery, Union Hospital, Tongji Medical College, Huazhong University of Science and Technology, Wuhan, China

**Keywords:** inferior vena cava (IVC), heterotopic ossification, retroperitoneum, mass, surgical resection

## Abstract

Masses of the inferior vena cava (IVC) are very diverse, most of which are thrombus and tumor thrombus, whereas heterotopic ossification of IVC has never been reported. Heterotopic ossification (HO) is the formation of mature lamellar bone outside normal bones and in soft tissues. Some researchers believe that HO is a manifestation of vascular calcification. Here we present a case of HO of the inferior vena cava (IVC) wall. A 68 year old female patient complaining hypertension and palpitation and diagnosed with a retroperitoneal mass was found to have a primary mass of the inferior vena cava wall during surgery. Histopathological examination after surgical resection revealed that the mass was mainly composed of mature bone tissues and hematopoietic tissues of bone marrow, there was no recurrence and the patient was symptom-free 15 months after the surgery. HO of the inferior vena cava wall is very rare, with large volume it can affect the circulation, and this case remind us that it can be cured by surgical resection.

## Introduction

Inferior vena cava (IVC) mass is a rare disease with a wide variety of histology and sources. Based on previous literature, IVC masses include thrombus, secondary tumor thrombus, and intravenous leiomyomatosis, primary IVC leiomyosarcoma, IVC phleboliths. The primary lesions of tumor thrombus can be from kidney, liver, and adrenal glands (1–5). Heterotopic ossification (HO) of IVC has not been reported before. Here we report a case of HO in the IVC that was diagnosed as a retroperitoneal mass before surgery, together with a literature review of HO and IVC masses.

## Case Presentation

**Figure 1:**
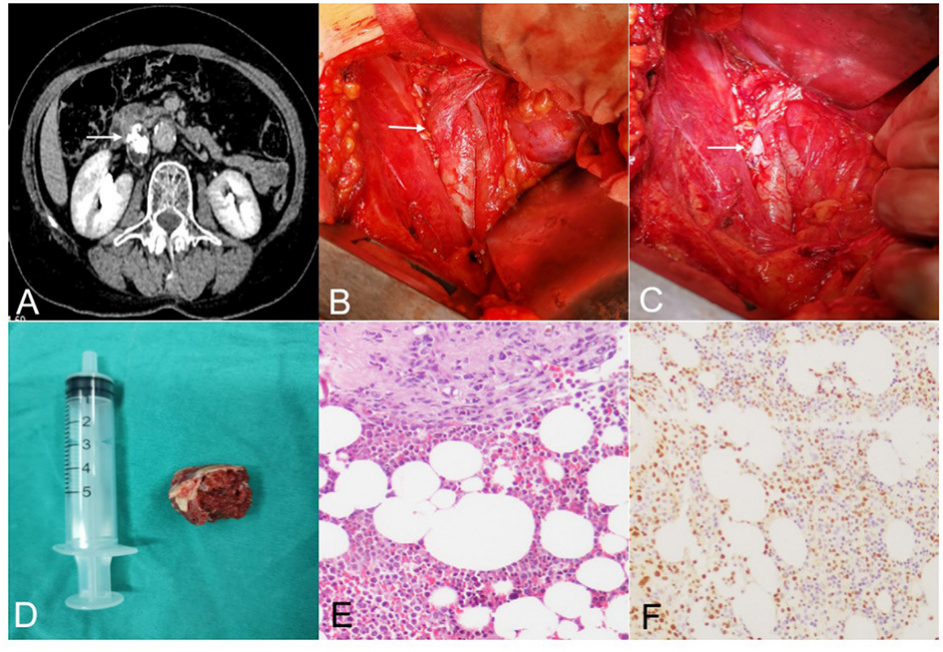
**(A)** Abdominal CT enhancement scan showed a mixed density image of about 22 * 28 mm in the IVC area with calcification and fatty density images [The lesion was indicated by the arrow in **(A–C)**]. **(B)** The mass originated from the wall of the IVC. **(C)** The mass had been removed, and the inferior vena cava was open and remains to be sutured. **(D)** The tumor was 2.5 cm in size, gray-red in color, solid and hard in texture. **(E)** Histopathological examination showed that mature bone fragmentation, hematopoietic tissue of bone marrow, and some free wall-like tissues were mainly seen under the microscope. **(F)** Immunohistochemical test showed positive ERG.

A 68 year old female patient went to her local hospital due to elevated blood pressure. The patient had paroxysmal hypertension with palpitation in recent years. The highest blood pressure was 200/110 mmHg. No history of trauma or interventional examination, and no history of injuries of the cord spine. Laboratory examination show serum calcium: 2.31 mmol/L, eGFR 90.7 ml/min/1.73 m^2^. A CT scan of the abdomen during hospitalization revealed irregular hyperintensity shadows in the inferior vena cava area and it was diagnosed as a retroperitoneal mass. The patient came to Wuhan Tongji Hospital in December 2019. Abdominal CT enhancement scan showed a mixed density image of about 22^*^28 mm in the IVC area and below the right renal vein, contained calcification and fatty density (Figure 1A). It was considered to be a retroperitoneal neoplasm compress the inferior vena cava. Later, the patient underwent laparoscopic retroperitoneal exploration, and it was found that the mass originated from the wall of the IVC. After conversion to open surgery (Figure 1B), we resected the mass and partial IVC wall adhered to the mass (Figure 1C), then repair the IVC wall with an extended polytetrafluoroethylene graft. The operation lasted 320 min. The patient started receiving low-molecular-weight heparin sodium treatment on the second day after surgery. The patient recovered and leave hospital 7 days after surgery.

On naked eye examination, the tumor was 2.5 cm in size, gray-red in color, solid and hard in texture (Figure 1D). Histopathological examination showed that mature bone fragmentation, hematopoietic tissue of bone marrow, and some free wall-like tissues were mainly seen under the microscope. Smooth muscle tissue was found in some of the wall-like tissues, of which there were two small pieces of tissue containing more epithelioid cells. Cytoplasmic vacuoles (immunophenotype suggests vascular endothelium) with interstitial hyaline degeneration which consistent with epithelioid hemangioendothelioma was also found in it (Figures 1E,F). Immunohistochemistry showed that epithelioid cells CD34, ERG, FL11, CD31 were positive. PCK, EMA, E-cad, TFE-3 were negative. Ki67 LI was about 2%. At the 15 month follow-up, CT scan showed there was no recurrence and the patient was symptom-free.

## Discussion

Heterotopic ossification (HO) is the formation of mature lamellar bone outside normal bones. At present, HO is generally considered as a complication after trauma, burns and nerve injury. The incidence of HO is 0.2–4% after burns, 10–53% after injury of the cord spine, and jumped to 90% after certain types of hip replacement or acetabular fractures (6–8). It is currently believed that the existence of osteoblast precursor cells, inducing factors, and appropriate microenvironment are the three conditions that must exist for the formation of HO (9). The origin of HO cell remains unclear. The research of D. Medici et.al have implicated vascular endothelial cells might be a potential source for HO progenitors (10).

Venous ossification is more special and rarer than ossification in other organs or tissues, and there are abundant studies shown that vascular calcification is a form of heterotopic ossification (11). Schmid et al. (12) Found that besides calcium phosphate, hydroxyapatite is also a mineral deposit in the atherosclerotic aorta. Then matrix vesicles were identified in calcified aortas, and microvascular pericytes were found to produce mineralized matrix *in vivo* (13). Later, BMP-2 was found expressing in calcified arteries (14). Some researchers found that calcified valves and vessels contain architecturally complex trabecular bone (15–18). These findings suggested that vascular calcification have same mechanism with osteogenic.

The clinical symptoms caused by HO in different tissues are variant. HO in the joint area can be presented as joint motion limitation (19), when it occurs to deep soft tissues such as muscles, compression symptoms can be manifested (20). When the mesentery is involved, it can show small bowel obstruction, symptoms such as nausea, vomiting, abdominal pain and bloating (21). In this case, the main symptoms were elevated blood pressure and palpitation, which might be related to compression of the IVC by the mass (22, 23).

HO of IVC could be misdiagnosed as many other diseases and requires careful identification. The histology and source of IVC masses are very diverse, their imaging findings varies, and their clinical manifestations are similar. Patients with IVC thrombus usually have congenital IVC abnormalities and deep vein thrombous of the lower extremity. Generally, patients with lower risk of deep vein thrombous of the lower extremity can exclude IVC thrombus (23). IVC leiomyomatosis mostly occurs in premenopausal women, often with uterine fibroids or a history of fibroid surgery. IVC leiomyomatosis caused by invasion of uterine fibroid cells. The tumor extends from the small vein to the IVC and even the right atrium. manifests as the disappearance of the flow-void signs in MR, and show different levels of uneven enhancement in enhanced scans (1). IVC leiomyosarcoma is more common in middle-aged women. The tumor originates from venous smooth muscle. CT can distinguish IVC leiomyosarcoma from IVC HO (24). The primary tumor can be found in all cases with IVC tumor thrombus, and it is not difficult to distinguish it from HO of IVC (3, 4). IVC Phleboliths are also very rare. Only one case is reported so far. Compared with IVC HO, venous stones are relatively regular in high density under CT and only adhere to the wall of the inferior vena cava (25). In addition to the lesions in the IVC wall and the lumen of the IVC, IVC HO also needs to be distinguished from retroperitoneal tumors that compress the IVC. Webb, E.M. founds that the disappearance of the inferior vena cava wall at the junction of the inferior vena cava and the tumor is an important indicator of the tumor's IVC origin (26).

Masses in the inferior vena cava are usually surgically removed or removed together with partial wall of the vena cava, especially for benign lesions, which can hopefully be cured. In this case, surgery was selected because the lesions were limited and the boundary were clear, and it had achieved favorable results.

## Conclusions

Ossification of IVC wall is a rare disease that has not been reported yet. Its pathological mechanism is related to heterotopic ossification and vascular calcification. Although it is a benign disease, it can affect patient's circulatory system if a large mass was formed, for single lesions with clear boundaries indicated by imaging examination, surgical resection can achieve good results.

## Data Availability Statement

The original contributions presented in the study are included in the article/supplementary material, further inquiries can be directed to the corresponding authors.

## Ethics Statement

The studies involving human participants were reviewed and approved by Medical Ethical Committee of Tongji Hospital of Huazhong University of Science and Technology. The patients/participants provided their written informed consent to participate in this study.

## Author Contributions

JT: writing-original draft and software. LZ: writing-review and editing, and supervision. MH: software and surgery. XZ: surgery and supervision. YW: review and editing. CY: surgery, funding acquisition, and writing-review and editing. ZH: surgery, project administration, and resources. All authors contributed to the article and approved the submitted version.

## Funding

This work was supported by the National Natural Science Foundation of China (Grant No. 81702989).

## Conflict of Interest

The authors declare that the research was conducted in the absence of any commercial or financial relationships that could be construed as a potential conflict of interest.

## Publisher's Note

All claims expressed in this article are solely those of the authors and do not necessarily represent those of their affiliated organizations, or those of the publisher, the editors and the reviewers. Any product that may be evaluated in this article, or claim that may be made by its manufacturer, is not guaranteed or endorsed by the publisher.
